# Supplementary material for: A Multiplex Analysis of Potentially Toxic Cyanobacteria in Lake Winnipeg during the 2013 Bloom Season
Source: Toxins (Basel). 2019 Oct 11;11(10):587. doi: 10.3390/toxins11100587 (PMC6832941; doi:10.3390/toxins11100587)
Supplement: Supplementary file 1 [file toxins-11-00587-s001.pdf]

Supplementary Materials: A Multiplex Analysis of Potentially Toxic Cyanobacteria in Lake Winnipeg during the 2013 Bloom Season

Katelyn M. McKindles, Paul V. Zimba, Alexander S. Chiu, Susan B. Watson, Danielle B. Gutierrez, Judy Westrick, Hedy Kling and Timothy W. Davis

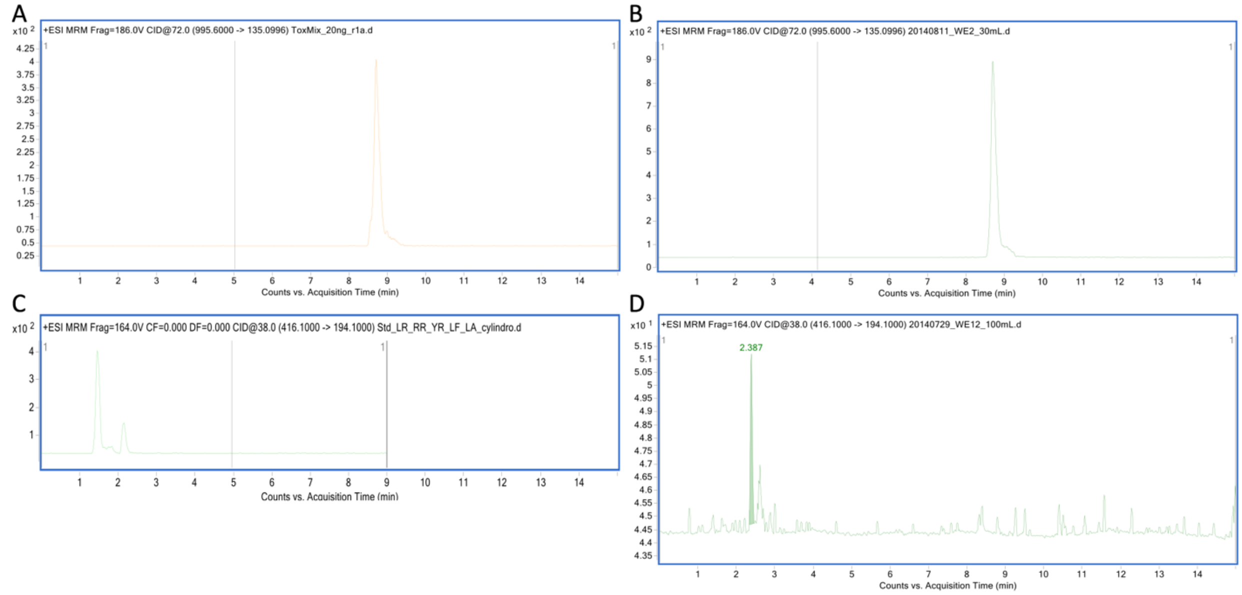

Figure S1. Example MS-MRM chromatograms for controls and sample hits for MC and CYN. (a) Chromatogram for MC-LR standard and (b) positive hit for MC-LR at site W2 sampled during summer 2013. (c) Chromatogram for CYN standard and (d) positive hit for CYN at site W12 sampled during summer 2013.
